# Paeoniflorin Protects against Acetaminophen-Induced Liver Injury in Mice via JNK Signaling Pathway

**DOI:** 10.3390/molecules27238534

**Published:** 2022-12-04

**Authors:** Xinyu Deng, Yubing Li, Xing Li, Zhenpeng Zhang, Shu Dai, Hefei Wu, Fangling Zhang, Qichao Hu, Yuan Chen, Jinhao Zeng, Xiao Ma

**Affiliations:** 1 State Key Laboratory of Southwestern Chinese Medicine Resources, School of Pharmacy, Chengdu University of Traditional Chinese Medicine, Chengdu 611137, China; 2 Department of Pharmacy, Chinese PLA General Hospital, Beijing 100039, China; 3 Hospital of Chengdu University of Traditional Chinese Medicine, Chengdu 610072, China

**Keywords:** paeoniflorin, acetaminophen, drug-induced liver injury, JNK signal, mitochondrial control of apoptosis

## Abstract

Background: Drug-induced liver injury (DILI), represented by acetaminophen (APAP), is a common cause of acute liver failure in clinics. Paeoniflorin (PF) has been proven to demonstrate a significant hepatoprotective effect. However, it is still unclear whether it can be a potential agent against hepatotoxicity induced by APAP. This study aimed to explore the preventive and therapeutic effects and mechanisms of PF on APAP-induced liver injury. Methods: Different doses of PF (50, 100, and 200 mg/kg) were given to C57BL/6 male mice for five consecutive days. After 12 h of APAP (250 mg/kg i.p.) treatment, blood and liver tissues were collected and isolated for detection. Results: The results showed that the therapeutic effects of PF on APAP mice were presented in the downregulation of the content of serum indices and significantly improved hepatic tissue edema and inflammatory infiltration. Meanwhile, PF reduces the level of the mitochondrial metabolic enzyme. Ulteriorly, it was found that PF has a downregulating effect on the apoptotic reaction and could inhibit the protein expression of CYP2E1/JNK signaling, which in turn reduces the damage of APAP. Conclusion: Our findings showed that PF acted as a protective agent against APAP-induced hepatotoxicity by inhibiting JNK-related signals, suggesting a novel insight into treating APAP-induced liver injury.

## 1. Introduction

Drug-induced liver injury (DILI) refers to a series of liver injury reactions caused by the excessive accumulation of active metabolites of drugs, herbs, and dietary supplements, which results in the most common reason for acute liver failure (ALF) in the USA and UK [1,2]. At the same time, a retrospective study on the incidence of DILI showed that the annual incidence of DILI in mainland China was 23.8 cases per 100,000 people, which was higher than that reported from Western countries, about 13.9–19.1 cases [3,4]. Due to its specificity, universality, and obstinacy, DILI, the typical injury reaction of ALF, is regarded as a liver disease with extremely high mortality, followed by viral hepatitis and liver fibrosis [5,6]. Therefore, DILI has become a major challenge for clinics and public health problems worldwide. The types of it are grouped into intrinsic and idiosyncratic in the clinic. Intrinsic DILI has a predictable incidence compared with idiosyncratic DILI and presents stable toxicity characteristics with the change of toxic dose, so it is often used as a model for DILI in preclinical studies [7]. Intrinsic DILI often occurs during clinical trials and preclinical testing for the development of new drugs, and dose-dependent hepatotoxicity can be anticipated and eliminated at an early stage [8]. Nevertheless, some drugs currently in the drugstore still have significant hepatotoxicity. One of the common drugs for fever and headache, acetaminophen exhibits obviously dose-dependent hepatotoxicity in overdoes or long-term use and causes more than 50% of cases of ALF in the West [9]. Overall, the study of the hepatotoxicity of APAP has attracted extensive attention.

Acetaminophen (APAP) (Figure S1A) is an effective non-opioid antipyretic and analgesic remedy that is generally highly tolerated by adults and juveniles at therapeutic doses. Under the circumstance of overdoes or long-term use of APAP, it leads to extensive liver injury and even ALF [10]. It is one of the most typical drugs that induce liver damage in the world. Meanwhile, abundant investigations have confirmed that the primary cause of APAP hepatotoxicity is an excessive accumulation of N-acetyl-p-benzoquinone imine (NAPQI), the chief reactive metabolite in APAP activated by cytochrome P450 enzymes, such as CYP2E1 [11,12]. Activated metabolites not only promote the activation of JNK but also initiate mitochondrial dysfunction by binding with proteins to form APAP-ADs, which induces mitochondrial-regulated apoptosis and endoplasmic reticulum (ER) stress and other processes, and ultimately damage the liver [13,14]. Thus, by reducing the accumulation of NAPQI and inhibiting the expression of JNK, it is expected to improve the liver injury induced by APAP. The therapies for liver injury caused by APAP are singular, and therapeutic drugs are scarce. The only clinically recognized drug, N-acetylcysteine (NAC), still has limitations, such as a narrow therapeutic window that restricts its use [15]. Although silibinin has been widely used in clinical liver diseases, its effect on APAP-induced liver injury is still not ideal [16]. Due to the extent and severity of the attack, it is urgent to explore new potential components and promote the clinical prevention and treatment of DILI.

**Figure 1:**
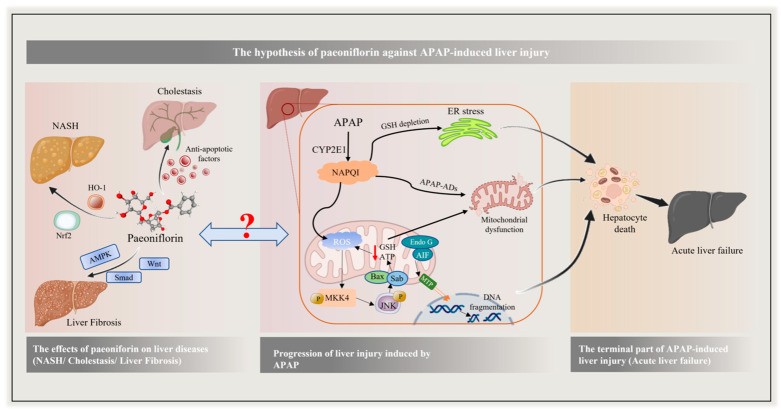
The hypothesis of paeoniflorin against APAP-induced liver injury. The pathogenesis of APAP is complex, which can induce hepatocyte death through multiple regulation, and then lead to acute liver failure. A large number of studies have shown that paeoniflorin can effectively improve many kinds of liver diseases. Based on this, we guessed that PF could be a choice against drug-induced liver injury caused by APAP.

Paeoniflorin (PF, Figure S1B) is the principal bioactive component derived from *Paeonia lactiflora* Pall. or *Paeonia veitchii* Lynch, which have been used to treat various liver diseases in the traditional medicine of China for many years [17]. Moreover, it has been verified, in recent research, that PF can protect the liver, prevent non-alcoholic fatty liver disease, relieve liver fibrosis, and inhibit hepatocellular carcinoma through the activities of anti-inflammatory, anti-oxidative stress, and anti-apoptosis [18,19]. Our studies also proved that PF demonstrates the significant effect of cholestasis alleviation via multiple pathways [20,21]. Undoubtedly, PF has a wide range of actions on the liver, yet there is still a lack of study on whether PF has a role in drug-induced liver injury, and we guessed that PF could be a choice against drug-induced liver injury caused by APAP (Figure 1).

As a result, an APAP-induced liver injury model in mice was established to testify to the preventive and therapeutic effects and safety of PF on drug-induced liver injury. Hepatic histopathology and indices of liver function were assessed to detect the states of liver damage. In addition, the mechanism was explored to illustrate the potential signaling pathway regulated by PF during DILI (Figure S2). The results could contribute to a better understanding of the disease and suggest novel insights into treating APAP-induced liver injury.

## 2. Results

### 2.1. Investigation of Experimental Conditions and PF Safety

In the initial liver injury, enzyme markers, such as ALT and AST, seeped into the blood from the liver, caused a sharp increase of levels in serum, and became the golden indicators of verification of liver injury [22]. Whereupon we considered them as a standard to confirm the experimental conditions, including the doses of APAP, the experimental period, the selection of positive drug, and safety. Compared with the control group, the levels of ALT and AST obviously rose in both the low (250 mg/kg) and high (400 mg/kg) dose APAP groups (*p* < 0.01), which shows that a DILI model could be established in two doses (Table S1). At the same time, in parallel with the low dose group, there was no change of biochemical indices in the high dose group (*p* > 0.05), indicating that the effect on APAP-induced liver injury was dose-independent, and the reason might be related to individual differences between mice. The liver injury induced by APAP overdose is rapid, violent, and typical, and the treatments are divided into acute and chronic therapies [9]. As a result, we planned to explore the appropriate experimental period at two days and five days. The results were shown that PF (200 mg/kg) could protect the liver after gavage for five days (Table S2). For the selection of a positive drug, the improvement of serum indices in the silibinin group was superior to the ursodeoxycholic acid (UDCA) group (Table S3). No significant effect was found between the control and HPF groups (Table S4 (*p* > 0.05)), which suggested there was no hepatotoxicity for PF (200 mg/kg).

### 2.2. Effect of PF on Macroscopic Pathological of the Liver

**Figure 2:**
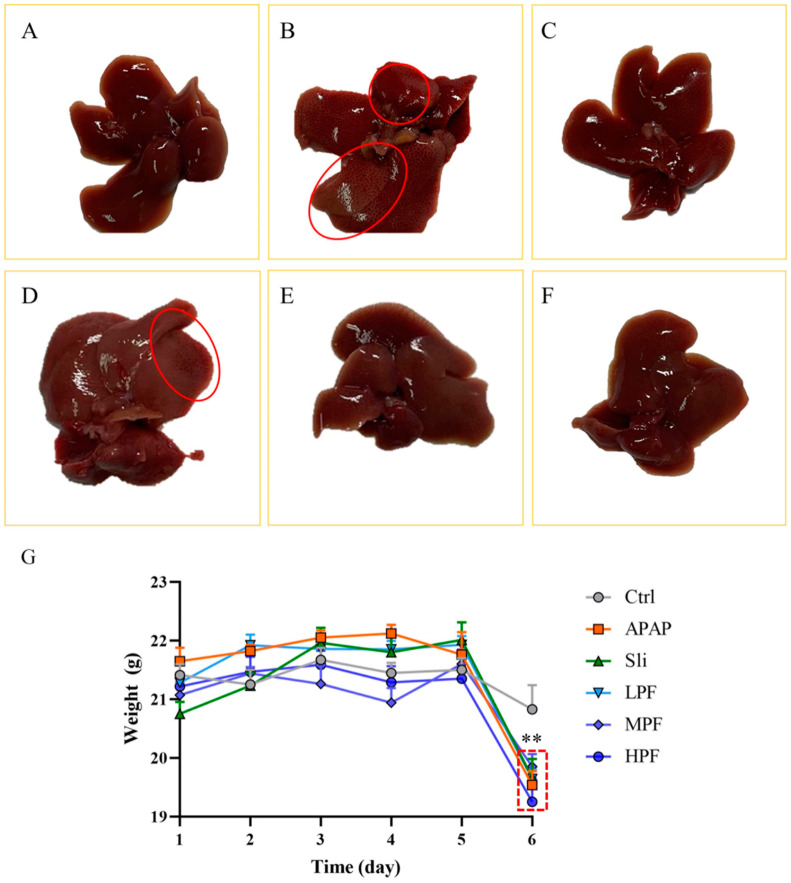
Effects of PF on macroscopic pathological changes of DILI mice. (**A**) The control group (Ctrl); (**B**) the APAP group (APAP); (**C**) the silibinin group (Sli); (**D**) the paeoniflorin low dose group (LPF); (**E**) The paeoniflorin middle dose group (MPF); (**F**) the paeoniflorin high dose group (HPF); (**G**) weight of mice during the experiment. The red circles indicate the lesions of the liver. ** *p* < 0.01, compared with Control group.

The different degrees of macroscopic pathological were observed to investigate the effect of PF against APAP. Intraperitoneal injection of APAP trigged liver granulation in the portal area and fibrosis in the margin compared with the control group (Figure 2A,B). In comparison with the APAP group, the damage signs of the HPF group and the silibinin group were obviously improved, while no effect was found in the LPF group (Figure 2C–F). As shown in Table 1, the liver index of the APAP group was on the increase (*p* < 0.05); conversely, the HPF and MPF groups could obviously reduce the liver index compared with the APAP group (*p* < 0.05), meaning liver swelling could be reduced by PF. During 5 days of gavage, the physiological changes in the mice were similar in each group, but the clinical signs of DILI were evidently exhibited after APAP was injected on the last day, such as loss of appetite and weight loss, and PF could relieve these symptoms (Figure 2G).

### 2.3. Preventive and Therapeutic Effects of PF on Liver Injury Caused by APAP

**Figure 3:**
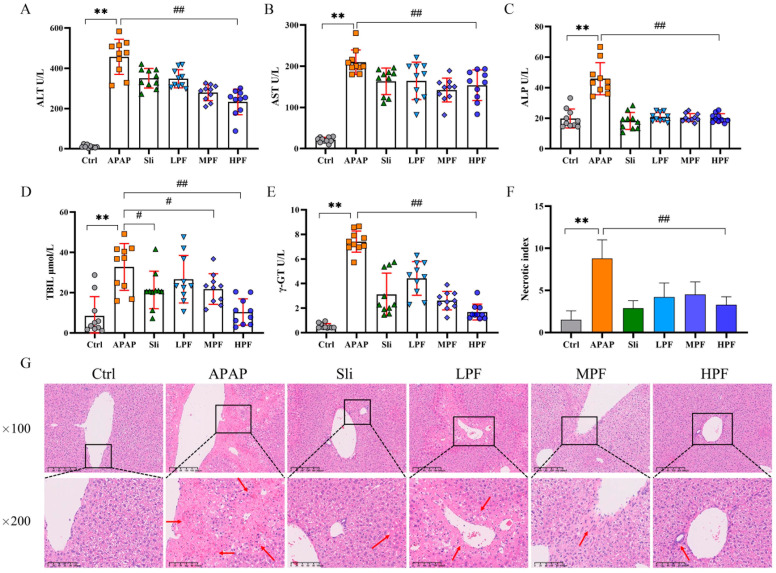
Preventive and therapeutic effects of PF on APAP-induced hepatic injury. Effect of PF on serum levels of ALT (**A**), AST (**B**), ALP (**C**), TBIL (**D**), and γ-GT (**E**) in liver injury caused by APAP. (**F**) Analysis of liver necrosis index. (**G**) Effects of PF on pathological liver changes in DILI mice (H&E staining, ×100, ×200). All data are expressed as mean ± standard deviation (SD). ** *p* < 0.01, compared with control group; ^#^ *p* < 0.05, ^##^ *p* < 0.01, compared with APAP group. The arrow points to the area of the liver lesion.

To verify whether there was an improvement of PF on injury, the contents of several specific markers in the liver, including ALT, AST, ALP, TBIL, and γ-GT, were measured. As observed in Figure 3A–E, the serum levels of those indices significantly increased in the APAP group compared with the control group (*p* < 0.01), meaning that the APAP of the liver injury had been successfully established. Compared with the APAP group, the HPF group (200 mg/kg) could signally decrease the levels of ALT, AST, ALP, TBIL, and γ-GT (*p* < 0.01), and several effects of the HPF group were even superior to the silibinin group. Meanwhile, the improvement of other serum indices in the MPF group (100 mg/kg) was like that in the HPF group, except for TBIL (Figure 3A–E). Additionally, the LPF group (50 mg/kg) could only affect ALT, AST, and ALP levels compared with the APAP group (*p* < 0.05) (Figure 3A–C), which hardly improved the TBIL (*p* > 0.05) (Figure 3D). The results of the serum indices indicated that PF could protect the liver from APAP-induced injury.

The critical evidence of the protective effects of PF on APAP-induced injury could be directly observed by histology. Mice in the control group showed intact hepatic lobules with a clear structure, abundant and tight, arranged around the central vein (Figure 3G). Corresponding to the results of serum indicators, the tissue structure of the liver from the APAP group was disorganized, many hepatocytes were necrotic, and the hepatocytes at the necrotic site were ruptured. The inflammatory factors infiltrated the portal area with obvious hepatic edema, and a large area of punctate necrosis could be clearly observed in a 100-fold microscope. Conversely, the mice in the PF and silibinin groups showed significant improvement in edema and necrosis. In particular, the €F in high dose (200 mg/kg) inhibited inflammation and alleviated APAP-induced damage. Those results are presented in Figure 3G. In addition, the number of necrotic per low power magnification was counted by blind method to calculate the liver necrosis index in order to further objectively evaluate the hepatic injury in mice. The trend of therapeutic effect, as expected, was consistent with that gained by serum indices (Figure 3F). Overall, these results showed that different concentrations of PF had different degrees of protective effect on the liver, illustrating the alleviative effects of PF on APAP-induced liver injury.

### 2.4. PF Prevents the Apoptosis in Mice Liver from APAP-Induced Injury

**Figure 4:**
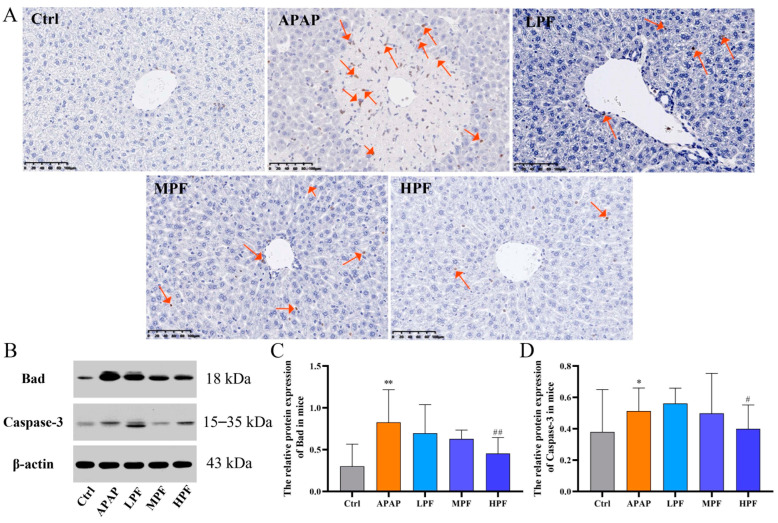
PF meliorated the damage of APAP through an anti-apoptotic effect. (**A**) The effects of PF on the apoptotic cell were verified by TUNEL staining. (**B**) Western blotting images of Bad and caspase-3 in mice. (**C**) The relative protein expression of Bad in mice. (**D**) The relative protein expression of caspase-3 in mice. All data were presented as mean ± SD. * *p* < 0.05, ** *p* < 0.01, compared with Control group; ^#^ *p* < 0.05, ^##^ *p* < 0.01, compared with APAP group. The arrow points to areas of elevated apoptotic expression.

Apoptosis, as a hot hypothesis for the pathogenic mechanism of DILI in recent years, has been reported in many studies [23,24]. Our research suggested that apoptosis from APAP induced in the early stage of liver injury could be inhibited by PF through the regulation of critical pro-apoptotic factors. The TUNEL staining used to detect apoptosis is shown in Figure 4A. Plenty of TUNEL-positive apoptotic cells concentrated on the damaged area were observed in the APAP group livers compared with the control group. The chromatin of the APAP group was shriven and marginalized, and the nuclear membrane even lysed. The positive markers greatly declined in the treatment groups, especially apoptosis in the portal area, and the state of the chromatin was similar to the control group. In addition, the hepatocytes in the HPF group had a clear morphology and obvious space and only a small amount of positive expression, suggesting that PF had a potential effect of relieving cell apoptosis. Moreover, the course of apoptosis is accompanied by changes to related apoptosis factors, such as the Bcl-2 and caspase families. Thus, the determination of protein expression levels of Bad and caspase-3 was applied to explore the mechanism of PF-improved DILI. In the Western blot test (Figure 4B), the assay results, as expected, showed an obviously inhibitory effect on PF in the expression of pro-apoptosis proteins. The HPF group notably downregulated the expression of Bad and caspase-3 (*p* < 0.01 or *p* < 0.05), the same effect was also shown in the MPF and LPF groups on the part of Bad, despite the alleviation of them on caspase-3 being hidden (Figure 4C,D). The findings indicated that PF pretreatment could prevent apoptosis for APAP-induced injuries, and the potential mechanism of it blocked the release of pro-apoptosis factors through the regulation of the mitochondrial apoptosis signal pathway.

### 2.5. The Protective Effect of PF on Liver Injury Related to CYP2E1/JNK Signal

**Figure 5:**
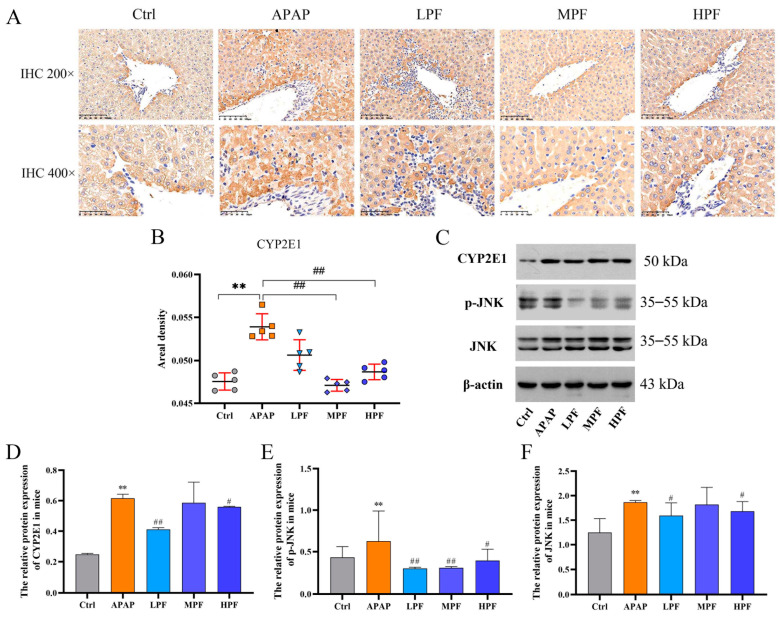
Effects of PF on the expression of CYP2E1/JNK signaling. (**A**) Immunohistochemical analysis of CYP2E1. (**B**) The areal density of CYP2E1. (**C**) The Western blotting images of CYP2E1, p-JNK, and JNK in mice. (**D**) The relative protein expression of CYP2E1 in mice. (**E**) The relative protein expression of p-JNK in mice. (**F**) The relative protein expression of JNK in mice. All data were presented as mean ± SD. ** *p* < 0.01, compared with control group; ^#^ *p* < 0.05, ^##^ *p* < 0.01, compared with APAP group.

In our experiment, the IHC revealed that APAP group samples expressed higher levels of CYP2E1 than control group samples. Correspondingly, the analysis of IHC showed the expression of CYP2E1 in the APAP group was distinctly elevated (*p* < 0.01), nevertheless, pretreatment with middle- and high-dose PF clearly reversed the liver damage of APAP stimulation compared with the APAP group (*p* < 0.01) (Figure 5A,B).

Western blotting was used to explore the level change in proteins affected by PF intervention (Figure 5C). PF in low and high doses could reduce the elevation of CYP2E1 triggered by APAP (*p* < 0.05 or *p* < 0.01) (Figure 5D). Beyond that, we also observed an obvious increase in JNK in DILI mice (*p* < 0.01), and a similar variation emerged from the phosphorylated JNK (p-JNK) compared with the control group (Figure 5E,F). However, the protein expression levels of these proteins declined in the PF groups. The LPF group exhibited conspicuously depressed protein expression levels (*p* < 0.01), while the HPF group showed weaker alleviation (*p* < 0.05) (Figure 5C,D). All the results suggested that not only the increase of CYP2E1 but also the overactivation of the JNK signal was demonstrated during the occurrence of APAP-induced liver injury. In the meantime, paeoniflorin effectively suppressed the content of CYP2E1 and restrained the JNK signal activation. Consequently, we considered that PF had a protective effect on damage generated by APAP through the CYP2E1/JNK signal.

### 2.6. PF Displays Favorable Affinity in CYP2E1 and JNK

**Figure 6:**
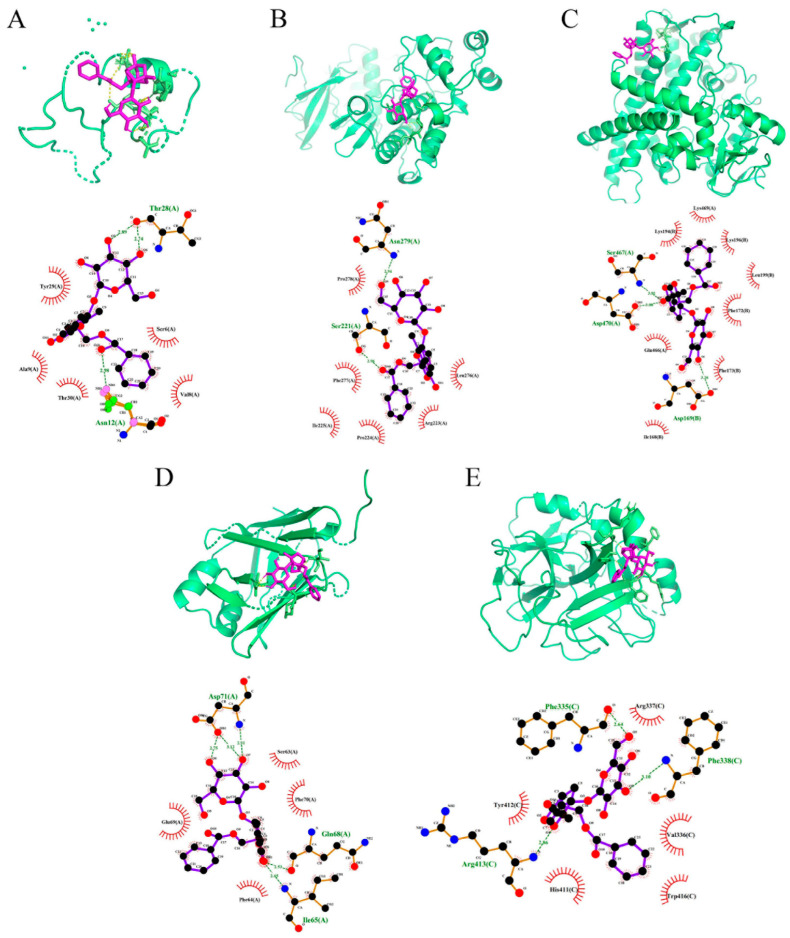
Molecular docking results of PF on main proteins. (**A**) PF-JNK; (**B**) PF-MAPK1; (**C**) PF-CYP2E1; (**D**) PF-Bcl-2; (**E**) PF-Bad.

According to the above results, we believe that the ameliorative effect of PF on APAP-induced liver injury is rooted in the regulation of CYP2E1, JNK, and its related downstream apoptosis signal. Therefore, we chose the main targets of JNK signaling as MAPK1 and JNK; the involved factors, such as Bcl-2 and Bad, were mediated by mitochondrial control of apoptosis as well as cytochrome P4502E to simulate binding PF to these proteins by molecular docking study. The docking fraction of PF and the proteins are shown in Table 2, and Figure 6 depicts an image of the docking of receptors and ligands after visualization.

The results demonstrated that PF could interact with CYP2E1, MAPK1, and Bad, and interact optimally with JNK and Bcl-2 (Figure 6A–E). PF formed one hydrogen bond with Asn279 and Ser221 in MAPK1 (Figure 6A). While in Figure 6B, the structure of PF could interact with Thr28 through two hydrogen bonds and one hydrogen bond with Asn12 in JNK. Figure 6C shows that PF formed one hydrogen bond with Gln68 and Ile65 in Bcl-2 and three hydrogen bonds with Asp71. In Bad, PF bound to Phe335, Phe338, and Arg413 through one hydrogen bond (Figure 6D). Figure 6E revealed that PF formed one hydrogen bond with Ser467, Asp470, and Asp169 in CYP2E1.

On the other hand, the lower the binding energy between the receptor and ligand is the superior their affinity is, and the more stable conformation they form. When the binding energy, which is less than 0 kcal/mol, indicates a possible binding activity between the ligand and the receptor, and less than −5 kcal/mol shows a good interaction for the receptor and the ligand [25]. The molecular docking results showed that these binding energies were all less than 0 kcal/mol, indicating that PF had an affinity with the MAPK1, Bad, and CYP2E1, a high affinity (less than −5 kcal/mol) with the JNK and Bcl-2 (Table 2). Among them, the docking of JNK and PF had the highest binding energy (−9.59 kcal/mol), and the docking of CYP2E1 and PF had the lowest binding energy (−3.59 kcal/mol). PF bound well to the JNK and Bcl-2, followed by Bad, with a poor affinity to CYP2E1 and MAPK1. By this token, we predict that JNK and its downstream apoptotic signal may be the key point for PF to execute a protective effect on the liver struck by APAP.

### 2.7. PF Ameliorates APAP-Induced Hepatotoxicity by Refraining Upregulation of CYP2E1/JNK Signal

**Figure 7:**
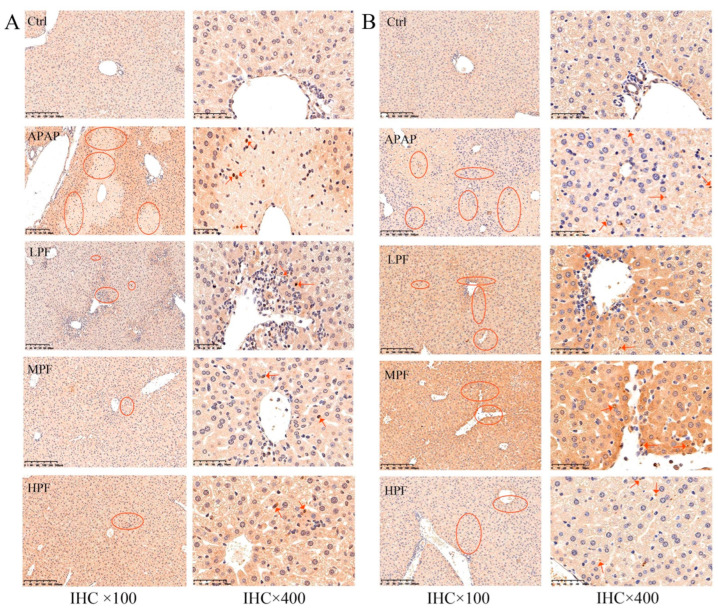
Expression of caspase-9 and caspase-3 was detected using IHC. (**A**) Expression of caspase-9 in different groups. (**B**) Expression of caspase-3 in different groups. The arrow points to the region of elevated protein expression, and the circles points to the area of elevated protein expression. (n = 3).

To further investigate the role of JNK and its activated apoptosis on PF-recovered APAP-induced liver injury, we detected the proteins of two major families involved in apoptosis regulation and sought the relationship between them. The expression of related apoptotic factors in IHC was investigated first. The results indicated that the APAP group samples expressed higher levels of caspase-9 than the control group, with a strong positive in the portal area of the liver, which meant obvious apoptosis appeared in the process of injury. Similarly, the control group samples expressed lower levels of caspase-3 than the triggered liver tissues. PF administration rebated the expression of caspase-9 and caspase-3, with a superior effect on the high dose (Figure 7).

**Figure 8:**
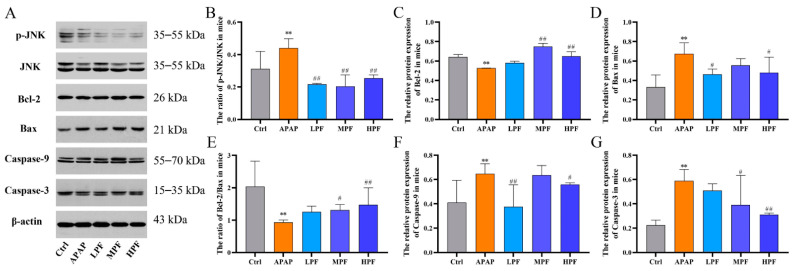
Effects of PF on the CYP2E1/JNK-signal-related protein expressions with APAP stimulation. (**A**) Western blotting images of p-JNK, JNK, Bcl-2, Bax, caspase-9, and caspase-3. The relative protein expression of Bcl-2 (**C**), Bax (**D**), caspase-9 (**F**), and caspase-3 (**G**). The ratio of p-JNK/JNK in mice (**B**). The ratio of Bcl-2/Bax in mice (**E**). All data are presented as mean ± SD. ** *p* < 0.01, compared with control group; ^#^ *p* < 0.05, ^##^ *p* < 0.01, compared with APAP group.

To further understand that PF-alleviated liver injury led to APAP by interfering with mitochondrial apoptotic signaling via JNK signal, the relative protein expression of p-JNK, JNK, Bcl-2, Bax, caspase-9, and caspase-3 in mice liver tissues were evaluated (Figure 8A). Compared with the control group, the protein expression of Bax, caspase-9 and caspase-3 were obviously enhanced after administration with APAP (*p* < 0.01), and Bcl-2, as the anti-apoptotic factor, the level of it was markedly curbed in the APAP group (*p* < 0.01). Conversely, PF could decrease the protein expression of Bax, caspase-9 and caspase-3 and increase the expression of Bcl-2, although the effect was inconsistent at different concentrations (*p* < 0.05 or *p* < 0.01) (Figure 8C,D,F,G). Moreover, an evident increase in the ratio of Bcl-2/Bax was observed in the PF intervention groups to different extents (*p* < 0.05 or *p* < 0.01), despite the decrease in the APAP group (*p* < 0.01) (Figure 8E). All these findings suggested that liver injury induced by APAP was associated with the regulation of mitochondrial apoptotic signaling, and this adverse condition could largely reverse by intervention with PF. In the aspect of CYP2E1/JNK signal, we investigated the expression of JNK and its phosphorylation products again, and the result of the ratio of two proteins manifested that the ratio in the APAP group was aggravated significantly compared to the control group (*p* < 0.01). Notably, the inhibition of PF on APAP-induced JNK activation was obvious. All doses of PF repressed the ratio of p-JNK/JNK (*p* < 0.01) (Figure 8B). In addition, combined with the changes in CYP2E1 protein expression mentioned above (Figure 5C), it could be confirmed that CYP2E1/JNK signal existed during APAP-induced injury in the liver, and this signal could further mediate mitochondrial apoptosis to induce hepatocyte death. Nevertheless, PF could reduce the level of CYP2E1 and restrain the activation of JNK, and then attenuate the expression of apoptosis factors to protect the liver destroyed by APAP. These results demonstrated that PF could alleviate APAP-induced hepatotoxicity by inhibiting mitochondrial apoptosis via down-regulation of the CYP2E1/JNK signal.

## 3. Discussion

Since it was first isolated from *Paeonia lactiflora* Pall, more and more reports have confirmed paeoniflorin’s, a monoterpene glycoside, multiple pharmacological activities, including cardiovascular protection, neuroprotection, tumor inhibition, and immunoregulation [26,27]. Meanwhile, PF has been widely used in a variety of hepatobiliary diseases because of its powerful hepatic protection [28]. By the regulation of HO-1, CYP2E1, and ROCK/NF-κB signaling, PF can improve non-alcoholic fatty liver disease [29,30]. The anti-apoptosis and anti-oxidative mechanisms of PF on toxic chemical-induced liver injury for regulation are mainly involved in MAPK/JNK and PI3K/Akt/Nrf2, as well as the mitochondria-dependent signals [31,32,33]. In addition, our previous study showed that PF had significant therapeutic effects on intrahepatic cholestasis caused by ANIT and extrahepatic cholestasis for the BDL model [21,34]. All these known results provide reasonable evidence for our hypothesis that PF can be a novel option for alleviating APAP-induced liver injury (Figure 1).

Acetaminophen is a representative drug to induce DILI and is also the common cause of liver failure in many countries [35]. Due to the similarity between the clinical symptoms of DILI and the effects of the model of APAP in rodents, these both resulted in weight loss, jaundice, and lack of appetite, so the construction of an APAP model in mice offers unique potential for exploring the pathogenic mechanisms of the APAP model in detail [36,37]. Thus, the role of PF in the pathogenesis and development of DILI was investigated by constructing an APAP-induced liver injury model in mice. We discovered the obviously typical DILI-like presentations after injecting APAP, and PF could improve them evidently, especially in high doses. In terms of physiological indicators, PF undoubtedly declined the increase of biochemical serum indices caused by APAP, while there was no distinct mitigating effect on TBIL except for a high dose. A possible reason is that TBIL is easily decomposed by light conditions, and errors occur in serum collection and determination of the index. The pretreatment of HPF and MPF also relieved the pathological changes in the hepatocytes. Moreover, the results of TUNEL staining showed that hepatocytes in PF groups had a clear morphology and only a small amount of positive expression in the administration of a high dose. Combined with the above findings, we thought that the protective effect of PF on liver injuries caused by APAP is related to apoptosis, and further studies are demanded to expound on the mechanism underlying the anti-apoptotic effect of PF.

It is reported that hepatocyte death is accompanied by persistent activation of JNK in APAP-induced damage of the liver. Once activated, JNK translocates to mitochondria, alters the permeability of the mitochondrial outer membrane, and then causes the generation of mitochondria-related apoptotic signals, subsequently resulting in hepatocyte death [38]. c-Jun-N-terminal Kinase is a member of the mitogen-activated protein kinase (MAPK) family, and the size of JNK proteins ranges from 46 to 55 kDa [39,40]. When hepatocytes are exposed to APAP, the levels of phosphorylated JNK elevate and migrate to the outer membrane leading to mitochondrial dysfunction and abnormal regulation of downstream apoptotic factors [41]. Our results were consistent with the reported study, and we found that PF visibly reduced the levels of p-JNK and JNK. The ratio of phosphorylated JNK to total JNK suggested that PF significantly downregulated the expression of JNK. In terms of JNK regulation, the reason why the effect of a low dose is superior to a high dose may be related to an error during experimental operation. Although there is a deviation between the effect of the mechanism and efficacy, the current results were analyzed with the original data. Meanwhile, related research has shown that there is a non-dose-dependent activation/inhibition effect between the drug and the target protein [23,42]. In addition, our results showed that PF could indeed inhibit JNK phosphorylation and ameliorate liver injury within the dose range studied (50–200 mg/kg).

Recruitment of multiple signals takes place in the process of liver damage induced by APAP, and mitochondria are the central point where the different signals responsible for initiation converge. These signals may lead to permeability conversion in the form of direct mitochondrial damage or possibly play indirectly through the activation of downstream apoptotic Bcl-2 family proteins [38,41]. Members of the Bcl-2 family are recognized for their common BCL-2 homology domain and their ability to regulate apoptosis. The family members can be divided into pro-apoptotic and anti-apoptotic proteins according to their activities, among which Bax and Bad belong to pro-apoptotic proteins, while Bcl-2 plays an anti-apoptotic role [43]. The Bcl-2 family, mainly located in the outer membrane of mitochondria, forms pro-apoptotic signals by altering the balance between Bcl-2 and Bax in APAP-induced liver injury [44]. Therefore, we measured the expression of Bcl-2, Bax, and Bad protein levels in the liver of mice suffering from APAP. As shown in our results, Bax and Bad were depressed under treatment with PF, while Bcl-2 was intensified. The ratio of Bcl-2 to Bax was shown to increase PF groups, revealing the anti-apoptotic effect. In addition, the Caspase family is another pathway to induce apoptosis. Caspases involved in apoptosis are divided into the initiator caspases, represented by caspase-9, and the effector caspases, including caspase-3 [45]. During the apoptosis, cytochrome C, located between the inner and outer membranes of mitochondria, is released from the mitochondria into the cytoplasm and binds to caspase-9 to form a cascade reaction signal, then activates caspase-3, and follows with apoptosis at last [46]. In our experiment, caspase activation could be significantly observed under the irritation of APAP, and pretreatment of PF effectively lessened the content of caspase-9 and caspase-3. At the same time, the inhibition of PF on the upregulation of caspase was also displayed under the IHC detection.

**Figure 9:**
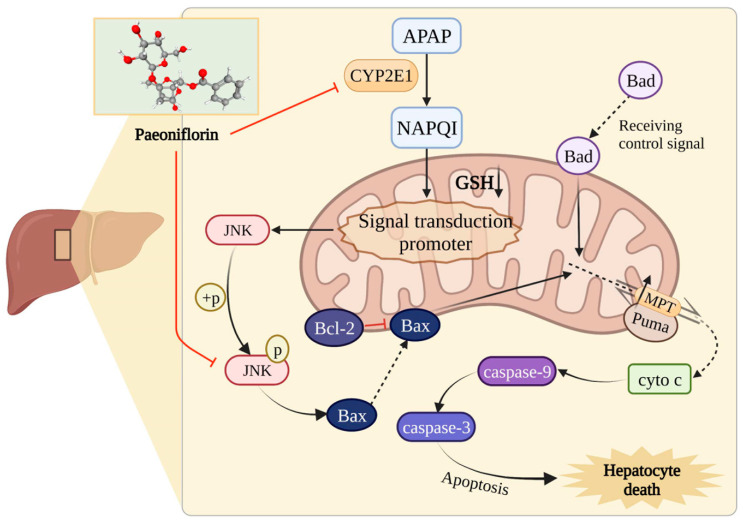
The mechanism diagram of PF on APAP-induced liver injury. The solid arrows show the metabolic course of APAP. The dotted arrows represent translocation of proteins. And the red arrows indicate inhibition of paeoniflorin.

The excessive accumulation of APAP intermediates produces ROS, which promotes the excessive activation of JNK, damages mitochondria, and results in the apoptosis and necrosis of hepatocytes [47]. Related research has reported the function of CYP2E1-targeting mitochondria, and increased mitochondrial CYP2E1 was found in livers during fasting [48]. Combined with the condition of liver poisoning caused by APAP, it can be assumed that CYP2E1 plays a role in mitochondria in liver injury induced by APAP overdose, thus synergistically stimulating JNK activation. In this context, our study showed that treatment of APAP in mice not only resulted in the activation of JNK and its downstream apoptosis signal but also aggrandized the content of CYP2E1. In addition, the prediction results of molecular docking showed that PF had a high affinity with JNK and Bcl-2 but poor binding ability with MAPK1, which is upstream of JNK, suggesting that JNK is the main target of PF against APAP. Although CYP2E1 had a poor affinity in prediction, the control of PF on CYP2E1 was seen by WB and IHC results. The possible reason is that the effect of PF on CYP2E1 activity is indirect. Hence, the results of this study indicated that APAP induces the activation of the JNK signal under the action of CYP2E1 and then initiates the mitochondria-dependent apoptosis signal, causing hepatocyte death ultimately. On the contrary, PF reversed the expression of JNK and CYP2E1, blocked the release of apoptotic factors, and improved liver function and pathological damage. PF acted as a protective agent against liver injury subjected to APAP by inhibiting mitochondrial apoptosis through down-regulation CYP2E1/JNK signal (Figure 9).

This study aimed to explore the therapeutic effects and mechanisms of PF in the treatment of APAP-induced liver injury. Therefore, the liver injury model induced by APAP (250 mg/kg) was first established, according to the reference [38]. The dose of PF was treated with the previous study of dosage in our research group [35]. Simultaneously, we selected male mice for the experiment because male mice are more sensitive to APAP, and estrogen could repair the hepatocyte [38]. Although NAC is currently a recognized antidote for APAP in the clinic, there are still side effects and a narrow treatment window, so silibinin, which has been used to treat liver diseases for a long time, was chosen as the positive drug [15,49]. In terms of mechanism, there is a dispute about the type of hepatocyte death caused by APAP in the literature in recent years. In this study, the detection time was 12 h after APAP injection, which was the early stage of cell death induced by APAP, and obvious apoptosis could be observed. With the aggravation of damage, apoptosis further reversely regulated the mitochondrial dysfunction, leading to cell necrosis, so broken hepatocytes could be observed in the results of the H&E and TUNEL staining. Nevertheless, there are still some limitations in this study. (1) The pathogenic mechanism of hepatotoxicity caused by APAP includes autophagy and oxidative stress in addition to the apoptosis regulated by the JNK signal, and it remains to be further studied whether PF can play a role in the treatment of APAP-induced liver injury through other pathways. (2) Our research has confirmed that PF can improve liver damage by regulating JNK signaling, but the specific protein-binding site of its role is unclear, which is a limitation of this study. (3) It needs to be combined with transcriptomic, proteomics, and other novel technologies for further exploration and verification. Collectively, our findings suggested that PF can improve APAP-induced DILI, possibly through regulation of the CYP2E1/JNK signal. Hence, our results, for the first time, demonstrated the pharmacological effects and mechanisms of PF in DILI. It may provide a new perspective on the potential of natural products to treat APAP-induced liver injury.

## 4. Materials and Methods

### 4.1. Reagents

Paeoniflorin (PF, purity: 98%; CHB190124) and acetaminophen (APAP, purity: 98%; CHB180228) were purchased from Chengdu Chroma-Biotechnology Company (Chengdu, China). Silibinin (Sli, capsule; 050603005), as a positive control, was acquired from TianJin Tasly Sants Pharmaceutical Co., Ltd. (Tianjin, China). PF and silibinin were dissolved in saline, while APAP was prepared in warm saline (66 °C) before use. Alanine transaminase (ALT), aspartate transaminase (AST), alkaline phosphatase (ALP), total bilirubin (TBIL), and γ-glutamyl transpeptidase (γ-GT) were provided by the Nanjing Jiancheng Bioengineering Institute (Nanjing, China). Other unspecified chemicals are analytical grade and were obtained from commercial sources.

### 4.2. Animals

Male C57BL/6 mice (20–22 g), aged 7–8 weeks, were purchased from Chengdu Dossy Experimental Animals Co., Ltd. (animal license No.: SCXK-(chuan) 2020-030). These mice were raised in the specific pathogen-free (SPF) environment of the Animal Experimental Center of Chengdu University of TCM. Prior to the experiments, the mice underwent adaptive feeding for 1 week in standard animal-rearing conditions with sufficient water and standard chow. The relative humidity was about 50%, and the room temperature was 25 ± 0.5 °C. In the meantime, the indoor lighting and darkness were alternated every 12 h. Up to 5 mice were placed in each plastic cage with bits of poplar bedding material, and the mice were randomly assigned in a blind fashion before receiving treatment. The details of the experimental set-up of mice are shown in Supplementary Figure S3. The approval of animal experiments was obtained by the ethical committee of Chengdu University of Traditional Chinese Medicine (No. TCM-2022-305). In addition, all experiments were performed in accordance with the guidelines and ethical codes.

### 4.3. Establishment of Experimental Model and Treatment

After acclimatization for 7 days, 6 groups of mice were assigned: the control group (Ctrl), APAP group (as model group, 250 mg/kg), silibinin group (Sli, as a positive group, 60 mg/kg), paeoniflorin low dose group (LPF, 50 mg/kg), paeoniflorin middle dose group (MPF, 100 mg/kg), and paeoniflorin high dose group (HPF, 200 mg/kg). Mice in all groups were given intragastric administration at a volume of 0.1 mL/10 g once a day for 5 consecutive days. The control and APAP groups received equal amounts of saline. The drug groups were given equal amounts of PF in different doses, and the positive group accepted an equal amount of silibinin. After the final administration, during the 12 h fasting period with free access to water, except for the control group, drug-induced liver injury was caused by intraperitoneal injection of acetaminophen (250 mg/kg) for the other groups. The control group accepted the same amount of normal saline. Twelve hours after the administration of APAP, the mice were killed by urethane. When the mice were sacrificed, the serum and liver tissues were collected for further biochemical and histological analyses.

### 4.4. Biochemical Analysis

The measurement of serum biochemical concentrations of ALT, AST, ALP, TBIL, and γ-GT was determined using a Varioskan flash-3001 (Thermo Scientific, Waltham, MA, USA) according to the manufacturer’s protocol.

### 4.5. Histological Hematoxylin–Eosin (H&E) Staining

The liver specimens were preserved in 4% paraformaldehyde and then dehydrated in a graded alcohol series. Then, liver specimens were fixed and embedded into paraffin blocks and were stained with hematoxylin-eosin (H&E) in sequence and placed under a microscope for histopathological study. Pathological sections were observed with the blind method under a light microscope, and the necrosis index was considered an indication of the degree of necrosis. The necrotic lesion index, meaning the number of necrotic zones per low-power magnification field (×100), was measured by counting the number of necrotic lesions in at least 10 fields of each slide under a low amplification [50].

### 4.6. TUNEL Apoptosis Assay

Apoptotic hepatocytes were labeled in situ using a TUNEL (terminal deoxynucleotidyl transferase (TdT)-mediated dUTP-fluorescein nick and labeling) peroxidase apoptosis detection kit according to the manufacturer’s instructions (Shanghai Roche Pharmaceuticals Limited, Shanghai, China). Slides of paraffin-embedded liver sections were deparaffinized and rehydrated. The fluorescein-labeled dUTP solution was used for staining and reaction in a dark and humid environment. After rinsing with PBS, 3,3′-diaminobenzidine (DAB) substrate was added and reacted at 15–25 °C for 10 min before rinsing with PBS again. Finally, the stained material was identified by a light microscope (Nikon, Tokyo, Japan), and the results of staining manifested different degrees of brown according to the stage of apoptosis.

### 4.7. Immunohistochemistry (IHC) and Quantification

Liver tissues were fixed with 4% paraformaldehyde for 10 min and then cleaned with PBS 3 times. Based on the above results, 3 samples from each group were selected for immunohistochemistry analysis. Briefly, samples were first decalcified by EDTA, followed by dehydration, transparency, and waxing. After embedding, samples were sliced into 4 μm and baked at 60 °C for 60 min and then stained. After rinsing the slices in tap water, they were repaired in an EDTA8.0 repair solution and then rinsed with distilled water. Slides were subsequently incubated in 3% H_2_O_2_ in PBS for 30 min at room temperature, followed by cleaning with PBS. Bovine serum albumin (BSA) blocking solution was dropped and incubated with slides for 30 min. Next, the slides were treated with indicated and diluted primary antibody overnight at 4 °C. The slides were incubated with a secondary antibody at 37 °C for 30 min after washing with PBS again. Subsequently, DAB staining and 3 washes with PBST were performed, followed by incubation in a hematoxylin working solution for 20 min. At last, the images were monitored by a microscope. All antibodies used are described in Supplementary Table S5. Antigen expressions in slides were observed and photographed by K-Viewer, and positive expressions were analyzed by Image J software. The ratio of the positive count area to the overall background area was used as the final result of positive expression analysis.

### 4.8. Protein Extraction and Western Blot (WB)

Total protein was extracted from liver tissue samples with ice-cold radioimmunoprecipitation assay (RIPA) buffer supplemented with phenylmethylsulfonyl fluoride (PMSF), protease inhibitor cocktail (Roche, Shanghai, China), and phosphatase inhibitor. The protein concentration was detected by using a bicinchoninic acid assay (BCA) protein assay kit (Solarbio, Beijing, China). After protein quantification using BCA, the protein lysates were transferred onto PVDF transfer membranes by polyacrylamide gel electrophoresis, then blocked and incubated to the corresponding antibodies, regarding β-actin as an internal control. The blots were visualized by scanning (Youke Excellence Biomedical Technology (Beijing) Co., Ltd.), and digital images and gray levels analysis were executed using the Gel Image System ver.4.00 (Tanon, China). All the details of the antibodies are shown in Table S5.

### 4.9. Molecular Docking

Molecular docking is a common technology for drug design that investigates the interaction and recognition of receptors and ligands. We used a molecular docking method to observe whether PF ameliorates APAP-induced liver injury by binding to related core proteins. The 3D structure of the target proteins was retrieved and downloaded from the protein database (PDB) (http://www.rcsb.org/, accessed on 16 May 2022), and we removed the water and replaced it with hydrogen in AutoDock4. The molecule structure of the drug was downloaded from the TCMSP and PubChem (https://pubchem.ncbi.nlm.nih.gov/, accessed on 16 May 2022) databases. Similarly, in AutoDock4, we took the same steps to set drugs as ligands. We imported the receptor and ligand PDBQT structures into AutoDock4 and defined the molecular docking range, as well as configured the operating methods and docking parameters. The PDBQT format was used to compute the minimal binding energy. The OpenBabelGUI software converted the composite PDBQT format to PDB format. Finally, for visualization, the composite PDB format file was imported using PyMol2.5 and LigPlot^+^ versions 2.2.

### 4.10. Statistical Analysis

The data were expressed as mean ± standard deviation (SD) and analyzed with the SPSS software program (version 25.0, SPSS Inc., Chicago, IL, USA), and the standard error of the mean was only used in calculating the weight curve of the mice. The conspicuous difference in Gaussian distributed data was determined by one-way ANOVA analysis. Graph Pad Prism software (version 8.0.0) was used to visualize the results, and Image J was used to analyze the positive expression of the figure. The differences were statistically significant when *p* < 0.05 and extremely significant when *p* < 0.01.

## 5. Conclusions

This study is the first to report the ameliorative effect and mechanism of paeoniflorin on liver damage induced by APAP, providing reference significance for the study of its preventive and therapeutic effects on APAP-induced hepatotoxicity. In summary, our results confirmed that paeoniflorin ameliorated the increase of liver function indexes, tissue damage, and activation of apoptosis caused by APAP through the CYP2E1/JNK signaling pathway. The findings of this study expand the application range of paeoniflorin in liver diseases. It also suggests a potential role in the treatment of DILI, which needs to be further investigated.

## Figures and Tables

**Table 1:** Effects of PF on organ indices in liver injury mice caused by APAP ($\bar{x}$ ± s, n = 5).

Group	Dosage (mg·kg^−1^)	Liver Index (mg·g^−1^)
Ctrl	None	30.78 ± 1.44
APAP	None	38.24 ± 4.09 *
Sli	60	33.43 ± 5.03
LPF	50	34.83 ± 7.69
MPF	100	31.45 ± 3.51 ^#^
HPF	200	32.27 ± 2.08 ^#^

* *p* < 0.05, compared with Control group; ^#^ *p* < 0.05, compared with APAP group.

**Table 2:** The binding energy of PF and core targets (kcal/mol).

Compound	Target	Target (PDB ID)	Target Structure	Affinity (kcal/mol)
Paeoniflorin	JNK	3NIR	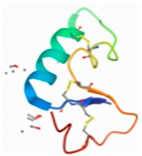	−9.59
	CYP2E1	2FDV	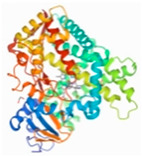	−3.59
	MAPK1	4ZZN	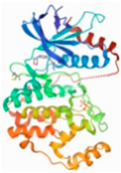	−3.66
	Bcl-2	3P6H	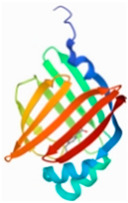	−7.30
	Bad	5PAX	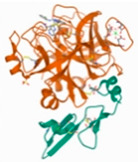	−4.33

## Data Availability

The data presented in this study are available on request from the corresponding author.

## Supplementary Materials

The following are available online at https://www.mdpi.com/article/10.3390/molecules27238534/s1, Figure S1: The chemical structures, Figure S2: The diagram of the experiment, Figure S3: Experimental procedure of PF against APAP-induced liver injury, Table S1: Effects of different doses of APAP on serum indices of C57BL/6 mice, Table S2: Effects of different time of experimental period on serum indices in C57BL/6 mice, Table S3: Effects of different varieties of positive drugs on serum indices in C57BL/6 mice, Table S4: Hepatotoxicity of paeoniflorin, Table S5: Details of antibodies used in this study.

## Abbreviations

ALP, alkaline phosphatase; ALT, alanine transaminase; APAP, acetaminophen; AST, aspartate transaminase; BSA, bovine serum albumin; CYP2E1, Cytochrome P450 family 2 subfamily E member 1; DAB, 3,3′-diaminobenzidine; DILI, drug-induced liver injury; H2O2, hydrogen peroxide; IHC, Immunohistochemistry; JNK, c-Jun-N-terminal Kinase; MAPK, mitogen-activated protein kinase; NAC, N-acetylcysteine; NAPQI, N-acetyl-p-benzoquinone imine; PBS, phosphate-buffered saline; PF, paeoniflorin; PMSF, phenylmethylsulfonyl fluoride; RIPA, radioimmunoprecipitation assay; SD, standard deviation; SPF, specific pathogen-free; TBIL, total bilirubin; TUNEL, terminal deoxynucleotidyl transferase (TdT)-mediated dUTP-fluorescein nick and labeling; γ-GT, γ-glutamyl transpeptidase.
