# Supplementary material for: Paeoniflorin Protects against Acetaminophen-Induced Liver Injury in Mice via JNK Signaling Pathway
Source: Molecules. 2022 Dec 4;27(23):8534. doi: 10.3390/molecules27238534 (PMC9739375; doi:10.3390/molecules27238534)
Supplement: Supplementary file 1 [file molecules-27-08534-s001.zip › molecules-2024602-supplementary.pdf]

Paeoniflorin protects against acetaminophen-induced liver injury in mice via JNK signaling pathway

Xinyu Deng[1], Yubing Li[1], Xing Li[2], Zhenpeng Zhang[1], Shu Dai[1], Hefei Wu[1], Fangling Zhang[1], Qichao Hu[1], Yuan Chen[1], Jinhao Zeng[3], Xiao Ma[1*]

[1] State Key Laboratory of Southwestern Chinese Medicine Resources, School of Pharmacy, Chengdu University of Traditional Chinese Medicine, Chengdu, 611137, China

[2] Department of Pharmacy, Chinese PLA General Hospital, Beijing, 100039, China

[3] Hospital of Chengdu University of Traditional Chinese Medicine, Chengdu 610072, China

[*] Corresponding authors: Jinhao Zeng, Email address: zengjinhao@cdutcm.edu.cn; Xiao Ma, Email address: tobymaxiao@cdutcm.edu.cn.

Supplementary File

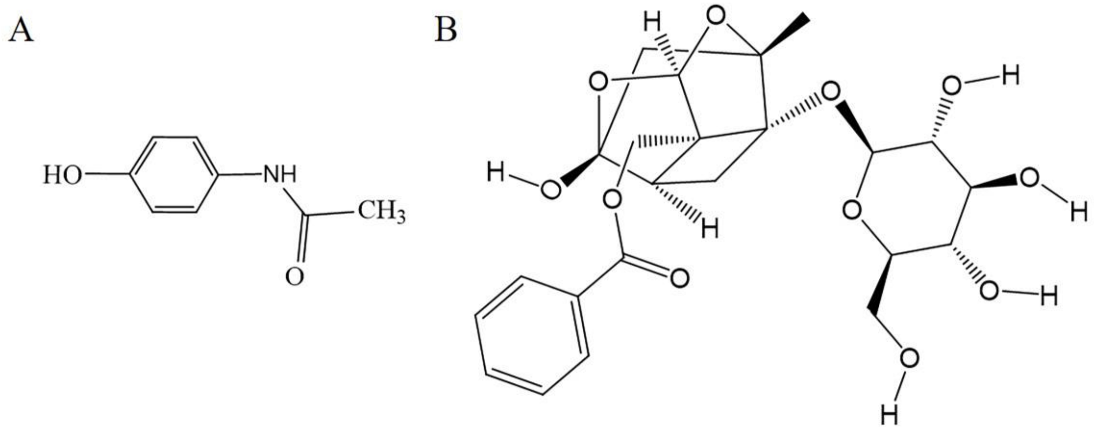

Figure S1. The chemical structures.

(A) the structure of acetaminophen. (B) the structure of paeoniflorin

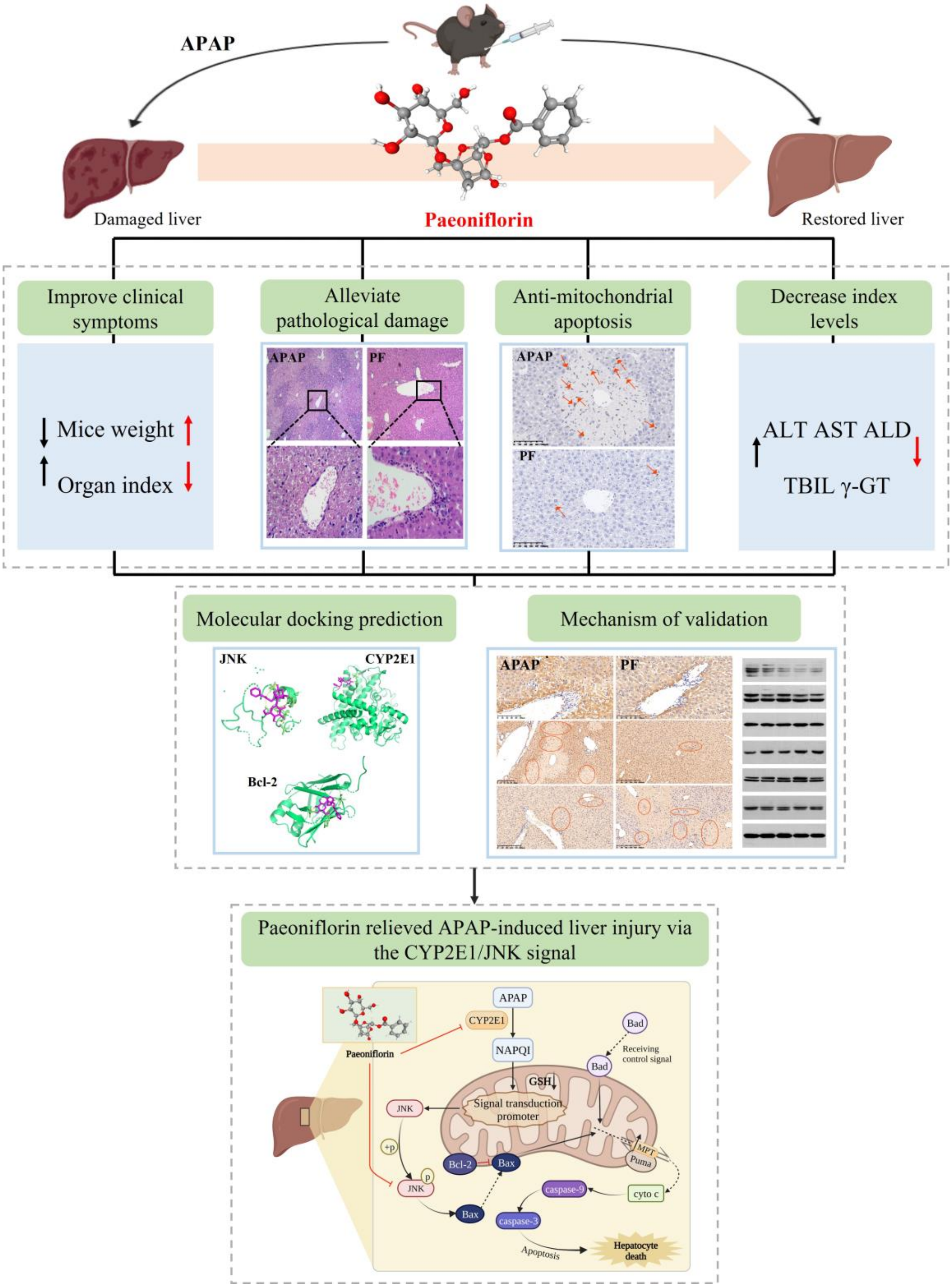

Figure S2. The diagram of experiment

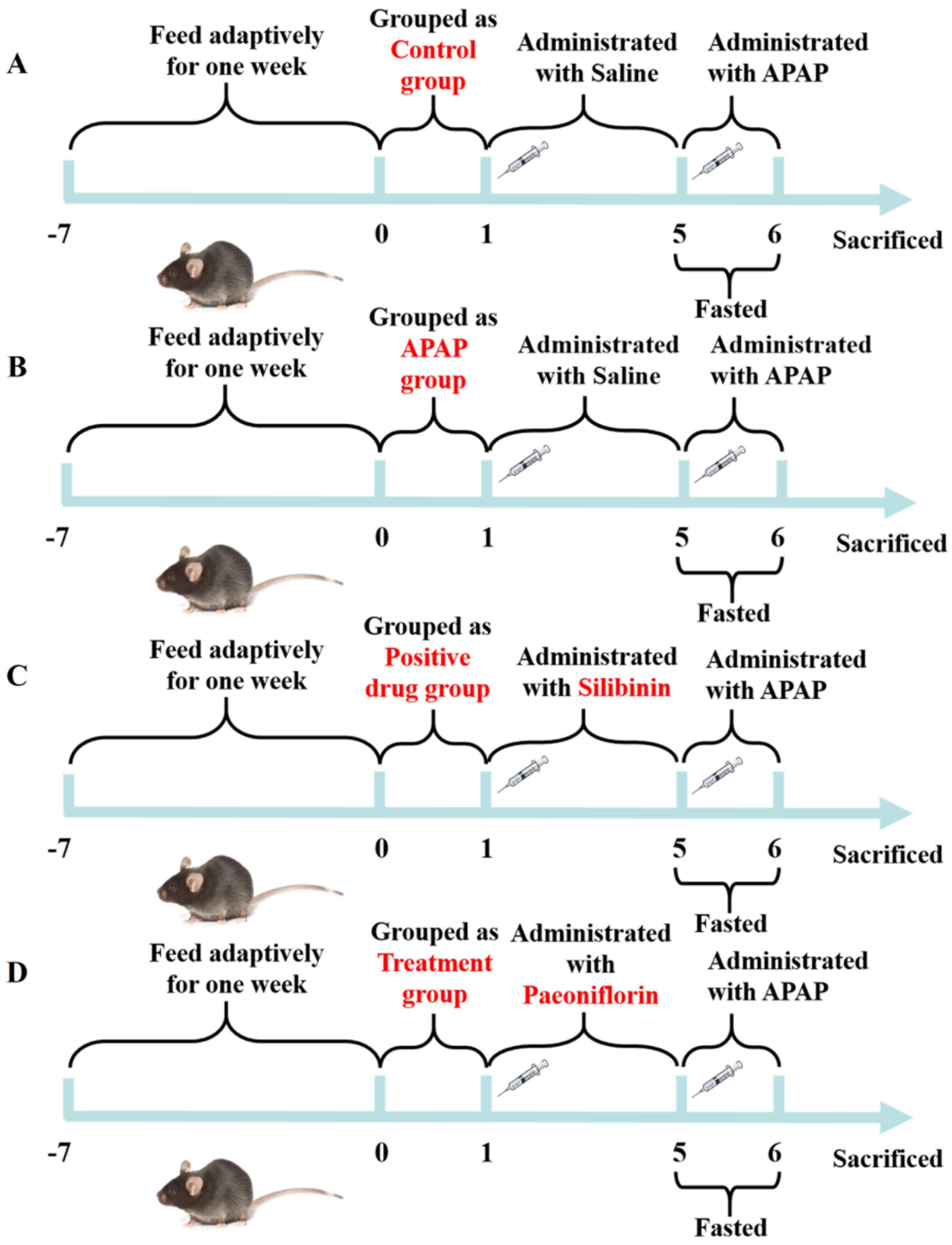

Figure S3. Experimental procedure of PF against APAP-induced liver injury

Table S1 Effects of different doses of APAP on serum indices of C57BL/6 mice

($\bar{x}$±s, n=6)

Group	Dosage (mg·kg^{-1})	ALT level (U·L^{-1})	AST level (U·L^{-1})
Ctrl	—	21.16±6.52	60.57±11.08
Low dose APAP	250	156.09±28.35**	184.63±42.81*
High dose APAP	400	169.89±21.24**	158.26±100.45*

Notes: $^{*}P$ < 0.05，$^{**}P$ < 0.01, compared with Control group

Table S2 Effects of different time of experimental period on serum indices in C57BL/6 mice

($\bar{x}$±s, n=6)

Period	Group	Dosage (mg·kg^{-1})	ALT level (U·L^{-1})	AST level (U·L^{-1})
	Ctrl	—	14.10±8.89	39.37±18.41
Two days	APAP	250	280.57±182.98**	136.35±73.42**
	HPF	200	402.88±36.38	162.94±44.71
	Ctrl	—	7.81±3.91	21.55±7.12
Five days	APAP	250	292.65±23.28**	187.33±14.65**
	HPF	200	178.22±57.44$^{\#\#}$	173.12±36.85

Notes: Ctrl: Control group; APAP: APAP group; HPF: High-dose group of Paeoniflorin. The periods of experiment are divided into the Two days and the Five days. **P < 0.01, compared with Control group； $^{\#\#}$ P < 0.01, compared with APAP group

Table S3 Effects of different varieties of positive drugs on serum indices in C57BL/6 mice

($\bar{x}$±s, n=5)

Group	Dosage (mg·kg^{-1})	ALT level (U·L^{-1})	AST level (U·L^{-1})
Ctrl	—	26.00±8.39	26.23±2.99
APAP	250	505.99±51.38**	212.59±15.73**
UDCA	55	487.75±61.34	202.64±18.14
Sli	60	309.02±24.94$^{\#\#}$	178.03±6.52$^{\#\#}$

Notes: Ctrl: Control group; APAP: APAP group; UDCA: Ursodesoxycholic acid group; Sli:

Silibinin group. **P < 0.01, compared with Control group; ## P < 0.01, compared with APAP group

Table S4 Hepatotoxicity of paeoniflorin ($\bar{x}$±s, n=5)

Group	Dosage ($mg \cdot kg^{-1}$)	ALT level ($U \cdot L^{-1}$)	AST level ($U \cdot L^{-1}$)
Ctrl	—	6.53±2.63	22.42±3.69
HPF	200	9.70±3.63	21.89±6.42

Notes: Ctrl: Control group; HPF: High-dose group of Paeoniflorin

Table S5 Details of antibodies used in this study

Antibodies	Host	Dilution	Catalogue	Manufacturer
**IHC**				
CYP2E1	Rabbit	1:100	19937-1-AP	Proteintech
Caspase-9	Rabbit	1:100	10380-1-AP	Proteintech
Caspase-3	Rabbit	1:100	19677-1-AP	Proteintech
**WB**				
JNK	Rabbit	1:2000	24164-1-AP	Proteintech
p-JNK	Rabbit	1:2000	80024-1-RR	Proteintech
CYP2E1	Rabbit	1:10000	19937-1-AP	Proteintech
Bad	Mouse	1:3000	67830-1-Ig	Proteintech
Bcl-2	Rabbit	1:500	26593-1-AP	Proteintech
Bax	Mouse	1:2000	60267-1-Ig	Proteintech
Caspase-9	Rabbit	1:300	10380-1-AP	Proteintech
Caspase-3	Rabbit	1:500	19677-1-AP	Proteintech
Beta actin	Rabbit	1:1000	TA-09	ZSGB-BIO
Goat anti-rabbit IgG(H+L), HRP conjugate		1:10000	111-035-003	Jackson
Goat anti-mouse IgG(H+L), HRP conjugate		1:10000	115-035-003	Jackson
